# Rupture of Mycotic Abdominal Aortic Aneurysm as a Result of Incompletely Treated Multiple Peripheral Mycotic Aneurysms

**DOI:** 10.3390/medicina60061007

**Published:** 2024-06-20

**Authors:** Lee Chan Jang, Dae Hoon Kim, Kwon Cheol Yoo

**Affiliations:** 1 Department of Surgery, Chungbuk National University College of Medicine, Cheongju-si 28644, Republic of Korea; 2 Department of Surgery, Chungbuk National University Hospital, Cheongju-si 28644, Republic of Korea

**Keywords:** mycotic aneurysm, abdominal aortic aneurysm, infective endocarditis, abdomen, lower extremity, case report

## Abstract

*Background*: A mycotic aortic aneurysm is a rare type of aortic aneurysm that can have disastrous outcomes. Most mycotic aneurysms originate from infectious sources, such as trauma, vegetation in the heart, and adjacent infectious sources. If a mycotic aneurysm is diagnosed, it should be treated simultaneously with the primary source of the infection. *Case Summary*: Treatment was performed for a mycotic aneurysm of the brachial artery that occurred suddenly during treatment for a fever for which the primary source of infection had not been confirmed. The workup revealed that a mycotic aneurysm of the brachial artery was the cause of the fever, followed by aneurysms in the abdomen and lower extremities and even vegetation in the heart that was not initially present. The patient declined to undergo treatment for personal reasons. After 5 months, it was revealed that the abdominal aortic aneurysm, which was initially considered normal aorta, was ruptured; however, the aneurysm was successfully treated. *Conclusions*: A peripheral mycotic aneurysm may be associated with multiple aneurysms. Appropriate diagnosis and complete treatments are necessary to prevent fatal consequences.

## 1. Introduction

A mycotic aneurysm, also referred to as an infected aneurysm, is a rare but fatal disease when it ruptures. Since mycotic aneurysms are usually found only during the autopsy in the absence of symptoms; their true incidence is often underestimated and difficult to determine [1]. In a previous study that analyzed autopsy results, mycotic aortic aneurysms constituted 3.3% of all aneurysms [2]. Even when a mycotic aneurysm is diagnosed and treated, mortality is reported in approximately 31% of cases; when it ruptures, mortality is reported in approximately 75% of cases [3]. The cause of mycotic aneurysms is unclear, and several theories have attempted to explain it through the analysis of a large number of cases [4].

We report a unique case of a peripheral mycotic aneurysm discovered incidentally; it was associated with multiple mycotic aneurysms that eventually developed infective endocarditis and a contained rupture of the mycotic aneurysm in the abdominal aorta.

## 2. Case Description

**Figure 1:**
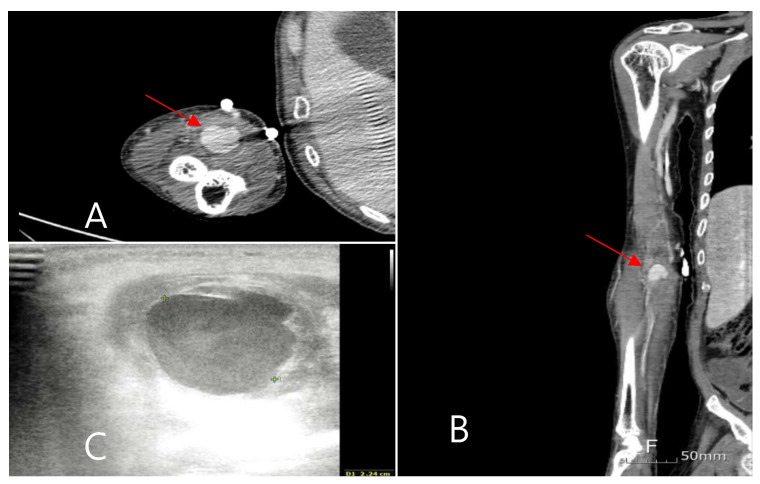
(**A**,**B**): Brachial artery mycotic aneurysm is observed in upper extremity CT. (**C**): Thrombosis and cellulitis around aneurysm are examined with Doppler.

A 63-year-old man presented to the emergency room with a fever of unknown origin that had not responded to antibiotic treatment for 2 weeks. In the nursing hospital, pneumonia and urinary tract infections, which commonly occur in hospitalized patients, were excluded during the examination, and the patient was diagnosed with an unknown fever. A combination of 4.5 g of piperacillin and tazobactam was administered intravenously every 6 h as a broad-spectrum antibiotic. The patient had been bedridden due to a traumatic subdural hemorrhage and a left corona radiata infarction. The patient also suffered a myocardial infarction that required aspirin administration. In the emergency room, the patient had no pain or tenderness in the body, and the lung sound was clear. The patient presented with mild leukocytosis, elevated C-reactive protein levels, and an elevated erythrocyte sedimentation rate. Rheumatoid diseases were excluded because of low complement levels, low immune antibody levels, and the absence of any related symptoms when examined by a rheumatologist. Five days after admission to the infection department, *Candida albicans* was cultured from the blood, and fluconazole was administered. Despite the administration of antifungal agents and antibiotics, a spiking fever of more than 40 °C, occurring two to three times a day, persisted. Echocardiography was performed to determine the fever’s cause and rule out infective endocarditis. A cardiologist performed transthoracic echocardiography; however, no vegetation was observed. The following day, transesophageal echocardiography was performed; however, vegetation was still not observed. After receiving conservative treatment for 2 weeks, the patient suddenly complained of pain in the right arm and localized swelling on the antecubital fossa during the third week of hospitalization. The pain was not accompanied by hyperemia or skin redness, but a tender, rigid, fixed, and palpable pulsatile mass was observed. Computed tomography (CT) of the upper limb was performed, revealing an aneurysm of the right brachial artery (Figure 1). Vascular ultrasonography revealed that the aneurysm contained a thrombus at the brachial bifurcation. Its maximum diameter was approximately 2.3 cm. The septic focus was determined to be a mycotic aneurysm of the brachial artery, and emergency surgery was performed. The mycotic aneurysm was completely removed, and bypass surgery was performed from the brachial artery to the radial and ulnar arteries using a bovine patch modified into a tube.

**Figure 2:**
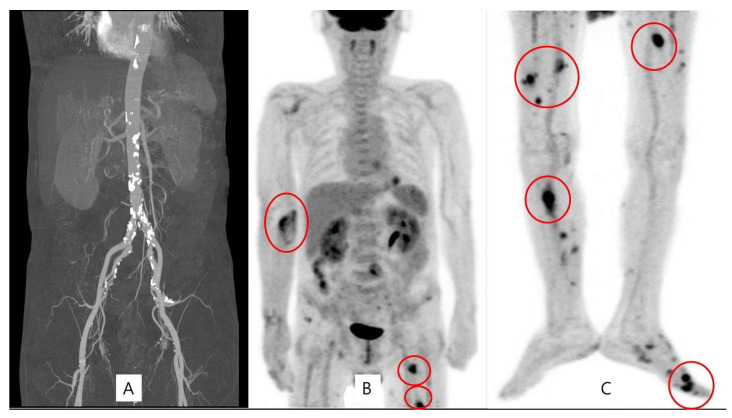
Normal aorta in abdominal CT (**A**) and multiple peripheral mycotic aneurysms were observed on the PET scan (**B**,**C**).

Although the symptoms in the arm disappeared, the spiking fever persisted. A positron emission tomography (PET) scan of the entire body was performed to evaluate the other origins of fever. It showed high uptake in multiple aneurysms in both lower extremities with normal aorta (Figure 2). A lower extremity CT scan was performed, wherein several aneurysms occluded by a thrombus were observed. A multidisciplinary meeting was held to determine the treatment strategy. Another cardiologist repeatedly performed echocardiography. Vegetation in the heart, which was not seen in the previous examination, was observed.

## 3. Final Diagnosis

The patient received a primary diagnosis of a peripheral mycotic aneurysm followed by one of infected endocarditis before receiving a final diagnosis: multiple mycotic aneurysms of the brachial artery and lower extremities with associated infective endocarditis.

## 4. Treatment

The patient declined to undergo additional surgical treatment for personal reasons; the systemic condition and fever improved with continuous administration of antibiotics and antifungal agents until discharge to a nursing home.

## 5. Outcome and Follow-Up

**Figure 3:**
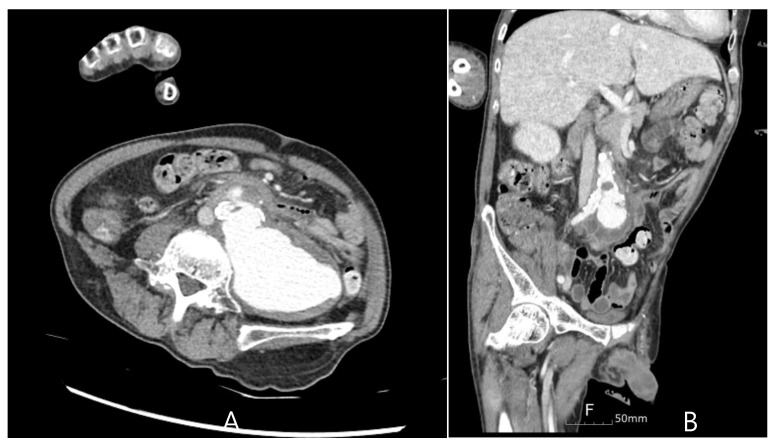
A ruptured abdominal aortic aneurysm was identified on a CT scan upon secondary admission. (**A**) Axial Plane, (**B**) Coronal Plane.

**Figure 4:**
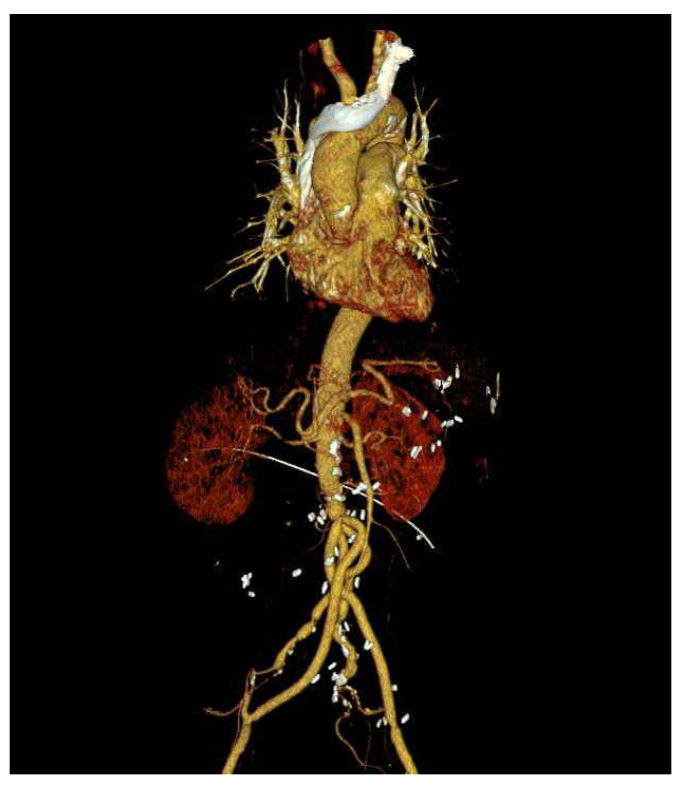
Postoperative CT scan of abdominal aortic aneurysm open repair.

Five months later, the patient was readmitted to the emergency room for recurrence of a fever of unknown origin. Leukocytosis was observed, but other laboratory examinations revealed no specific findings. To evaluate the focus of the fever, chest and abdominal CT scans were performed, as shown in Figure 3. The findings revealed a contained rupture of the mycotic abdominal aortic aneurysm (AAA). The patient underwent emergency AAA repair using an antibiotic-soaked graft. No complications were observed on the CT scan performed 6 months after surgery (Figure 4), and the patient has been living without complaints of other symptoms over a 1-year follow-up.

## 6. Discussion

Mycotic aneurysms have a high risk of rupture, leading to morbidity and mortality [3,5]. The most common site of mycotic aneurysms is the intracranial artery, followed by the abdominal aorta. The other prevalent sites of mycotic aneurysms are the femoral and brachial arteries because they are common injection sites for drug abusers [4]. However, our patient was bedridden and was not immunocompromised or a drug abuser. Direct seeding from adjacent sources of infection or trauma and septic emboli from infective endocarditis can cause mycotic aneurysms of the peripheral arteries [4].

It has previously been reported that 50–70% of blood cultures have identified the following infectious agents: *Salmonella* species, *Staphylococcus aureus*, *Bacteroides fragilis*, *Escherichia coli*, and *Pseudomonas aeruginosa* [3,6,7]. Contrary to other reports, *C. albicans* was identified in our patient, and broad-spectrum antibiotics were administered prophylactically along with antifungal agents.

The brachial artery was an unusual location for the development of mycotic aneurysms [6], given that the patient was not a drug abuser or an immunocompromised individual. Moreover, the patient had no history of surgery or infective endocarditis during the early stages of evaluation. According to previous literature, mycotic aneurysms have a primary infectious source, such as trauma, psoas abscess, osteomyelitis, or endocarditis [3]. In the present case, the origin of the mycotic aneurysm was not confirmed. Adjacent sources of infection, such as lytic destruction or osteomyelitis [8], were not identified. In addition, endocarditis was initially excluded using transthoracic and transesophageal echocardiography. We speculated that the mycotic aneurysm of the brachial artery might not have initially arisen alone but might be one of several mycotic aneurysms of the extremities. Septic emboli from several incompletely treated peripheral mycotic aneurysms were thought to have eventually induced the vegetation in the heart, which may have contributed to the delayed rupture of the mycotic abdominal aortic aneurysm.

The treatment of a peripheral mycotic aneurysm involves in situ reconstruction or extra-anatomic bypass after removal of the mycotic aneurysm, along with long-term antibiotic treatment [9]. Most reported cases have been solitary mycotic aneurysms, and complete resection of the mycotic aneurysm could be achieved. However, our patient had multiple mycotic aneurysms, and his condition was not ideal for extensive surgical removal of all; therefore, long-term antibiotic treatment was administered after the surgical resection of the most symptomatic aneurysm. Several peripheral aneurysms suspected of being mycotic were controlled using antibiotics; however, the patient’s abdominal aortic aneurysm seemed to have deteriorated, resulting in a contained rupture. The patient underwent AAA repair using a rifampin-soaked graft.

Several case reports have documented cerebral mycotic aneurysms caused by infective endocarditis [10,11,12,13,14,15,16,17,18]. However, this case differs from other reports in that infective endocarditis developed while treatment for multiple peripheral mycotic aneurysms was underway and was followed by a mycotic abdominal aortic aneurysm rupture.

## 7. Conclusions

According to previous reports, mycotic aneurysms are caused by a primary infectious source, such as trauma, osteomyelitis, or endocarditis. In this case, the mycotic aneurysms, which occurred in various peripheral arteries without any other causative infectious lesions, had catastrophic consequences and eventually led to the contained rupture of the abdominal aortic aneurysm. Fatal consequences might be inevitable when mycotic aneurysms are not completely treated.

## Data Availability

The original contributions presented in the study are included in the article. Further inquiries can be directed to the corresponding author.
